# GWAS integrating maternal and child genetics reveals novel risk-modifying loci for fetal alcohol spectrum disorders (FASD)

**DOI:** 10.21203/rs.3.rs-9950101/v1

**Published:** 2026-09-10

**Authors:** Vivek Ruhela, Corina Lesseur, Ariadna Cilleros-Portet, Sandra W. Jacobson, Joseph L. Jacobson, Ernesta M. Meintjes, Neil C. Dodge, Tugba Akkaya-Hocagil, H. Eugene Hoyme, Haoxiang Cheng, Jia Chen, Ke Hao, Maya A. Deyssenroth, Giuseppe Tosto, R. Colin Carter

**Affiliations:** 1 Department of Neurology, Taub Institute for Research on Alzheimer’s Disease and the Aging Brain, and Gertrude H. Sergievsky Center, Columbia Vagelos College of Physicians and Surgeons, New York, New York, USA.; 2 Department of Environmental Medicine and Public Health, Icahn School of Medicine at Mount Sinai, New York, NY, USA.; 3 Department of Psychiatry and Behavioral Neurosciences, Wayne State University School of Medicine, Detroit, Michigan, USA.; 4 Department of Human Biology, Faculty of Health Sciences, University of Cape Town, Cape Town, South Africa.; 5 Department of Psychiatry and Mental Health, Faculty of Health Sciences, University of Cape Town, Cape Town, South Africa.; 6 Department of Biostatistics, School of Medicine, Ankara University, Ankara, Turkey; 7 Sanford Children’s Genomic Medicine Consortium, Sanford Health, Sioux Falls, South Dakota; Department of Pediatrics, USA.; 8 University of Arizona College of Medicine, Tucson, Arizona, USA.; 9 Department of Genetics and Genomic Sciences, Icahn School of Medicine at Mount Sinai, New York, NY, USA.; 10 Department of Environmental Health Sciences, Mailman School of Public Health, Columbia University, New York, NY, USA.; 11 Departments of Emergency Medicine and Pediatrics, Institute of Human Nutrition, Columbia Vagelos College of Physicians and Surgeons, New York, New York, USA.

## Abstract

Fetal alcohol spectrum disorders (FASD) exhibit wide phenotypic variability not explained by prenatal alcohol exposure (PAE) alone. While genetic factors may modify vulnerability, there are no prior FASD genome-wide association studies (GWAS). We performed genome-wide single-marker and gene-based analyses of two South African prospective birth cohorts (Cape Town Longitudinal Cohorts (CTLC); n=328 mother-child dyads) and the US Collaborative Initiative on Fetal Alcohol Spectrum Disorders cohort (CIFASD n=429 children only). We examined main genetic and gene-by-PAE interaction effects on working memory, recognition memory, height, and FASD diagnosis using single-marker and gene-based analyses and the Haplotype-based Transmission Disequilibrium Test (HTDT), which integrates maternal-child genetics. Genome-wide genotype array data were used; TOPMed-imputed data were used to refine results. Risk-modifying genes were then tested for differential mRNA expression based on PAE in placenta and child blood. Gene*PAE interaction gene-based analyses identified *STX6* associated with height-for-age Z-scores (*p*=2.2×10^−6^) and *FOXD3* with recognition memory performance (*p*=4.7×10^−6^) in children. Single-marker analysis detected one child SNP approaching significance (*rs2833924*, *p*=8.8x10^−8^) with Gene*PAE interaction for working memory performance. CTLC+CIFASD meta-analysis in children identified one significant locus (*HTR1E; p_meta_*=2.75×10^−6^) with Gene*PAE interaction for working memory performance. The HTDT test in CTLC imputed data identified three haplotypes associated with FASD diagnosis risk: *COP1 (*p_adj_=0.029*)*, *WWOX* (*p_adj_*=2×10^−4^), *TMEM38B (p_adj_= 0.019)*. *WWOX*, *STX6*, and *TMEM38B* were differentially expressed in placenta and/or child blood based on PAE. In this first-ever genome-wide investigation of maternal and child genetics in FASD, we identified multiple biologically plausible risk-modifying loci for FASD neurobehavioral, growth, and FASD diagnosis outcomes.

## 1. INTRODUCTION

Prenatal alcohol exposure (PAE) is the are among the most common preventable causes of neurodevelopmental disabilities worldwide [1, 2]. Despite prevention efforts, 14% of women in the US (10% worldwide) continue to drink during pregnancy [3]. Prevalence estimates of fetal alcohol spectrum disorders (FASD), the diagnostic term for children with adverse effects of PAE, range from 1-5% in the US and Europe to 13.6-30.6% in other parts of the world [1, 2]. FASD manifest a broad range of cognitive/behavioral deficits and growth restriction; teratogenic effects of alcohol have been reported in every organ system [4-8]. Individuals with FASD exhibit lifelong learning disabilities, behavioral problems, and social challenges, which affect individual quality of life and place substantial demands on healthcare, education, and social systems [9]. Fetal alcohol syndrome (FAS) is characterized by distinctive facial dysmorphology, small head circumference, and pre- and/or postnatal growth restriction [6]. Partial FAS (PFAS) includes individuals with characteristic facial anomalies and one of: small head circumference, growth restriction, or neurobehavioral deficits. Children with the most prevalent form of FASD, alcohol-related neurodevelopmental disorder (ARND), are the most difficult to identify, as they lack the characteristic facial dysmorphology, but still have cognitive impairment that is often as severe as children with FAS or PFAS.

Children with prenatal alcohol exposure (PAE) exhibit remarkable variability in the severity of neurocognitive deficits, which is not explained by dose and timing of alcohol exposure alone; only 10-15% of children with heavy PAE develop FASD, while 4-10% develop full fetal alcohol syndrome (FAS) [10]. Genetic factors make large contributions to FASD phenotypic variability in both human and animal studies. Animal models have shown that different genetic strains exhibit different vulnerability to the teratogenic effects of alcohol [11]. In human sibling studies, monozygotic twins were almost fully concordant for FASD diagnosis, while 36-44% of dizygotic twins and 41 and 22% of full- and half-sibling pairs were concordant, respectively[10]. In prospective longitudinal birth cohorts in Cape Town, South Africa, we recently found that although ancestry was not associated with cognition in the absence of PAE, maternal and child ancestry were associated with altered risk of FASD outcomes [12]. For example, for every 1% increase in maternal or child East Asian ancestry, the odds of an FAS/PFAS diagnosis decreased by 15%.

Maternal genetic profiles are needed to identify women at risk for having a child with FASD in need of intensive screening and prevention efforts. Child profiles are needed to identify children at high risk for FASD for whom treatment interventions could be started early in life when they are most effective. Novel risk-modifying genetic loci may also elucidate mechanisms underlying the teratogenic effects of alcohol and identify novel therapeutic targets. However, despite three decades of research, few genetic determinants of FASD phenotypic variability have been identified. The dearth of prior knowledge regarding genetic influences in FASD may be due to prior analyses being limited to candidate genes [10]. Candidate gene approaches in human studies have identified important potential risk-modifying polymorphisms in genes involved in alcohol metabolism [13-17], choline transport [18], retinoic acid pathways [19], and N-glycosylation [20]. However, candidate gene studies require *a priori* knowledge of influential genes, which is often lacking and sometimes incorrect. By contrast, GWAS studies may identify completely novel genes with pathophysiologic significance. However, to our knowledge, none have been employed in FASD studies to date. This may be due in part to genome-wide interaction screens are especially power-intensive, making such studies particularly difficult in FASD research given the relatively small sample size of cohorts with high levels of PAE and deep phenotyping and the relatively low exposure levels in larger cohorts [10, 21]. Innovative approaches are thus needed.

To our knowledge, prior studies of genetic influences in FASD have not examined both maternal and child genetics. Examining both maternal and child genetics is critical given the impact of both on the intrauterine environment and shared genetics between the mother and child. While some genes may be relevant only in the mother (e.g., alcohol metabolism genes), others may be relevant only in the child (e.g., genes impacting neurodevelopment), and others may be relevant in *both* mother and child (e.g., genes in nutrient metabolism, placental development, etc.). Furthermore, when examined in isolation, a variant identified as risk-modifying in the child may actually be a proxy for the mother’s genotype, or vice versa, due to shared genetics.

In this study, we sought to identify genetic loci in the mother and child that alter fetal vulnerability to PAE in genome-wide analyses from two South African prospective longitudinal birth cohorts and the US-based Collaborative Initiative for FASD (CIFASD) clinical cohort. To our knowledge, this is the first genome-wide study of genetic influences in FASD and the first to integrate maternal and child genetics. We first employed classic single-marker genome-wide screens, separately for mothers and children. To reduce multiple testing burden, we then employed a gene-based approach, separately for mothers and children. Recognizing that these approaches do not examine maternal and child genetics simultaneously, we applied the Haplin Transmission Disequilibrium Test (HTDT), which disentangles maternal and child genetic contributions to disease risk.

## 2. MATERIALS AND METHODS

### 2.1 Cape Town Longitudinal Cohort (CTLC) description

Data are from two prospective longitudinal cohorts designed to examine the developmental effects of PAE: one recruited from 1999-2002 (“CTLC 1”) [8, 22]; the other from 2011-2015 (“CTLC 2”) [23, 24]. Alcohol consumption at recruitment was ascertained in timeline follow-back interviews [25, 26]. Women were invited to participate if they averaged ≥1.0 oz (30 mL) absolute alcohol (AA)/day (≈1.67 standard drinks) or reported binge drinking (based on National Institute on Alcohol Abuse and Alcoholism (NIAAA)) binge definition at the time of recruitment: ≥2.5 oz (75 mL) AA/occasion for CTLC 1; (≥2.0 oz (60 mL) AA/ occasion for CTLC 2). Women who abstained or drank only minimally (<1 oz (30 ml) AA/day on average with no binge episodes) were enrolled as controls. Exclusion criteria at recruitment included maternal age <18 years, HIV infection, multiple gestation pregnancy, and pharmacologic treatment for medical conditions. Infant exclusion criteria included major chromosome anomalies, recognizable malformation syndromes (other than FASD), neural tube defects, very low birthweight (<1500 g), and extreme prematurity (gestation <29 weeks). DNA samples were unavailable for both mother and child for 25 mother-child dyads with heavy exposure and 20. Nine participants failed initial quality control procedures (see 2.3 below). The final cohorts for the analyses presented here included 128 mother-infant dyads (76 with heavy PAE, 52 controls) from CTLC 1; 200 mother-infant dyads (120 with heavy PAE, 80 controls) from CTLC 2 (Figure 1).

Each woman provided informed consent in the mother’s preferred language (Afrikaans or English). Women reporting alcohol or drug use were counseled by our staff about the risks of prenatal alcohol and drug use, were encouraged to stop or reduce their alcohol and/or drug use and were offered referrals to substance use treatment programs.

### 2.2 Prenatal alcohol exposure, covariates, and phenotypic characterizations

In timeline follow-back interviews [25] administered at recruitment and again in the 3^rd^ trimester (CTLC 1) or 4 and 12 weeks after recruitment (CTLC 2), each mother was asked about her alcohol, smoking, and drug use on a day-by-day basis during the past 2 weeks, with recall linked to specific daily activities. Maternal age and Hollingshead socioeconomic (SES) scores were obtained in maternal interviews [27].

Four *a priori* outcomes were chosen to capture FASD clinical spectrum: working memory, recognition memory, height-for-age (Z-scores), and FAS/PFAS diagnoses. Working memory and recognition memory assessments were the California Verbal Learning Test-Children’s version (Pearson Clinical Assessments, USA) at age 10 years in CTLC 1 and the Wide Range Assessment of Memory and Learning, 2^nd^ Edition (Pearson Clinical Assessments, USA) at age 7 years for CTLC 2. Height was measured by trained staff, and age- and sex-specific Z-scores calculated [28] (ages 1 and 5 years in both cohorts and ages 9 and 16 years in CTLC1). In diagnostic clinics in 2005, 2009, 2013, and 2016, children were independently examined for growth and FASD-related dysmorphology by expert FASD dysmorphologist HE Hoyme MD and other trained FASD dysmorphologists, all blinded to PAE and prior diagnoses. FASD diagnoses were determined in case conferences using the Hoyme criteria [6]; consistent with these criteria, “cases” for neurobehavioral outcomes were defined as scoring <1.5 SD below the mean for unexposed children and <10^th^ percentile for height.

### 2.3 Genotyping Assays

Please see Supplementary Materials for sample collection and DNA extraction details. DNA from whole blood or saliva samples were assayed using the Illumina (San Diego, USA) Infinium Expanded Multi-Ethnic Genotyping Array (MEGA^EX^), which contains >2 million SNPs and covers 65.7% of GWAS catalog SNPs. Genotyped data were cleaned with standard QC measures using PLINK (v1.9). In brief, SNPs were excluded if Hardy-Weinberg equilibrium *p*-value <1e-06 and missing call rate <95%; individuals were removed if genotype missingness ≥2%. Principal component analysis (PCA) was conducted using *king* software [29] to account for variance in population stratification and to identify unrelated set of samples (defined as kinship <0.09).

To identify genetic outliers, we performed PCA analysis using PLINK (version 1.9.10) [30, 31] on individual SNP genotype data and compared the distribution of the top principal components (PCs). Individuals having PC values beyond the range of (Mean of all PCs) ± (four standard deviations) were excluded (4 children and 16 mothers). Covariate outliers were identified employing the Rosner test [32]: for a) cohort, b) mothers’ age, c) SES, d) cigarette exposure, and e) weekly gestation, only SES and cigarette exposure showed outliers and were thus adjusted. Values exceeding the upper outlier threshold identified by the Rosner test were winsorized by replacing them with the corresponding upper-bound cutoff value, a commonly used robust statistical approach for minimizing the influence of extreme observations while preserving sample size and distributional stability [33]. 256 mother-child dyads had complete genotype data for the Haplin transmission disequilibrium test (HTDT; see below). Rare SNPs (those with minor allele frequency (MAF) <5%) were filtered out. The pre-processing workflow is shown in Supplementary Methods (Figure S1).

### 2.4 Gene Expression Assays

Please see Supplementary Materials for sample collection and RNA extraction details. Using bulk-tissue RNA extracted from placental and child blood (age 5 years for CTLC1; 9 years for CTLC2) samples, libraries were sequenced to a depth of approximately 50 million 100-bp paired-end reads per sample. Raw sequencing reads were pre-processed to remove adapter sequences and filtered to eliminate low-quality reads (Phred score < 20). Reads were aligned to the GRCh38.v33 human reference genome using the STAR aligner, and gene-level quantification was performed. Only protein-coding genes with raw counts >10 in at least 50% of the samples were retained, resulting in a final expression matrix of 14,867 genes. One placenta sample showing >5% estimated maternal cell contamination, identified through prior integration of maternal-infant genotyping and transcriptomic data, was excluded. After additional quality control filtering, including removal of low-RNA Integrity Number (RIN) samples, the final dataset included RNA-seq data from 62 high-quality placental samples and 308 high-quality blood samples.

### 2.7 Imputation

To increase power to detect signals associated with SNPs that might be missing from the genotype array, we performed genotype imputation for CTLC data using the TOPMed Imputation Server [34]. Variants with an imputation quality score (R^2^) below 80% were filtered out and the resulting high-quality data were converted into PLINK format for processing and further genetic association and analytical workflows (Figure 1).

### 2.6 CIFASD cohort dataset

The CIFASD data resource includes data from 269 children with PAE (defined as >4 drinks/occasion at least weekly or >13 drinks/week) and 269 normotypically developing unexposed (<1 drink/week and <2 drinks/occasion) children with child genetic, demographic, and phenotypic data [35]. Fully de-identified data are available to researchers after application to and approval by the CIFASD steering committee. 107 individuals were removed due to lack of exposure data. No genetic outliers were detected based on PCA. Children ages 7-18 yr were recruited from 2007-2012 and 2014-2017 from clinical and research sites at San Diego State University, UCLA, University of Minnesota, and Emory University. Exclusion criteria included: significant head injury with loss of consciousness, significant or psychiatric (e.g. psychosis) disability that would preclude participation and, for the exposed group, other known causes of cognitive deficiency (e.g. congenital hypothyroidism, neurofibromatosis, chromosomal abnormalities). 17% of exposed children have full FAS. The cohort is 32% African American, 19% White, and 49% Hispanic. Outcomes examined included age-/sex-specific height Z-scores, working memory (Behavioral Rating Inventory of Executive Function-Preschool (BRIEF-P)), recognition memory (Differential Ability Scales (2^nd^ Edition; DAS-II) Recall of Designs (within-cohort *Z*-score) or, if DAS-II was not administered, Cambridge Neuropsychological Test Automated Battery Delayed Matching to Sample (within-cohort *Z*-score)), and clinical FAS diagnosis.

Samples were genotyped on the OmniExpress genome array (Illumina) or Multi-Ethnic Genotyping Array [36][37], and imputed using the Michigan Imputation Server [34] using the HG19 build 1000 Genomes Phase 3, b37 reference panel [38]. The final dataset consisted of 4,000,362 SNPs with MAF ≥ 0.05, genotype rate ≥ 0.99, and Hardy–Weinberg equilibrium *P* ≥ 0.000001 (Figure 1).

### 2.8 Statistical Analysis

All statistical analyses were performed using R (version 4.3.1) [39]. The complete workflow overview is shown in Figure 1.

For working memory, recognition memory, height-for-age (Z-scores), we employed the following model:

$$outcome∼PAE+genotype+PAE^{*}genotype+covariates.$$

For FAS/PFAS diagnosis – since it can only occur in the setting of PAE - we limited the analyses to children with PAE and employed the following model:

Diagnosis∼genotype+covariates.

Based on our prior studies, covariates included cohort (CTLC1 vs CTLC2), maternal age at conception and SES for all outcomes and, additionally for height-for-age and FAS/PFAS diagnosis, prenatal cigarette exposure (cigarettes/day) and weeks gestation at delivery. In CIFASD, SES, maternal age at conception, and prenatal cigarette exposure data were not available. All analyses included the top three PCs.

Separately for mothers and children in CTLC and children in CIFASD, we employed the genome-wide efficient mixed-model association using the GEMMA [40] for single-marker analyses and the Genome-wide Complex Trait Analysis (GCTA) [41] for gene-based analyses (see Supplementary for full options employed). For binary outcomes, GEMMA models the phenotype using a linear mixed-model approximation rather than a generalized linear mixed model with a logistic link function. For continuous outcomes, linear mixed models with an identity link function were employed. To increase statistical power and evaluate consistency of genetic effects across cohorts, we performed a meta-analysis using the MetaSoft framework under the Han and Eskin random-effects model, which accounts for heterogeneity in effect sizes between cohorts while maintaining statistical power under heterogeneous effects [42]. Meta-analysis was performed separately for each neurobehavioral and growth outcome across cohorts.

For mother–child dyads, we conducted haplotype-based association testing using the Haplotype-based Transmission Disequilibrium Test (HTDT) implemented in the Haplin R package [43, 44]. Haplin applies maximum likelihood estimation to phased haplotypes and accommodates uncertain phase and missing parental genotypes. We analyzed 4-SNP sliding-window haplotypes under a multiplicative dose–response model, which estimates per-allele dosage effects. For each haplotype, Haplin produces separate maternal and child effect estimates, including relative risks (RR) with 95% confidence intervals, single and double dose p-values, and population haplotype frequencies, allowing us to distinguish whether associations arise from maternal genotype and fetal genotype patterns. Because sliding-window haplotypes overlap extensively, the resulting tests are not independent, and standard FDR procedures (e.g., unweighted Benjamini–Hochberg) can be overly conservative. Therefore, we applied a frequency-weighted Benjamini–Hochberg (BH) correction, where haplotypes with higher population frequency receive proportionally greater weight. This approach improves statistical power while maintaining false discovery rate control by incorporating independent covariate information (here, haplotype frequency) into the multiple-testing procedure, as implemented in the Independent Hypothesis Weighting (IHW) framework [45].

For genes identified in the above analyses, differential gene expression was examined using DESeq2 [46] based on exposure (yes vs. no) was examined, adjusting for surrogate variables, the covariates described above, and gene expression-inferred cell-type proportions for all cell types with mean proportion >2% in the cohort [47] using the model:

Geneexpression∼exposure+tissue-specificcell-typecomposition+technicalcovariates

## 3. RESULTS

### 3.1 Sample characteristics.

As we have previously reported [12], mothers were 27.4 years old at conception on average, and heavy drinking mothers were older than controls (Table 1). Heavy drinking and control mothers completed 8-10 years of school, on average, with heavy drinking women averaging fewer years of school than controls. Heavy drinking mothers in both cohorts exhibited a weekend-binge pattern of drinking, averaging 7.0 standard drinks/occasion on 1.4 days per week in CTLC 1 and 6.7 standard drinks/occasion on 1.4 days per week in CTLC 2. Most control women abstained from drinking during pregnancy, with 12 women reporting infrequent, light consumption (no binges (i.e., <4 standard drinks/occasion), with drinking occasions <2/month for 10, <1/week for 2). Cigarette smoking was more common among heavy drinking women than controls in CTLC 1, but the number of cigarettes smoked per day was similarly low in both groups. 14.5% of heavy drinking women vs. 3.8 % of controls in CTLC 1 and 24.2% of heavy drinking women vs. 10.0 % of controls in CTLC 2 reported prenatal marijuana use. 11.7% vs. 15.0% of control women in CTLC 2 reported prenatal methamphetamine use; no women in CTLC 1 reported methamphetamine use, as this was uncommon in the area in the early 2000’s. Methaqualone, cocaine, and opiate use were not reported by any participant. Most women (82.0% in CTLC 1; 86.5% in CTLC 2) delivered at term. 47.4% of exposed children in CTLC 1 and 30.8% of children in CTLC 2 met diagnostic criteria for FAS or PFAS. The CIFASD database has been extensively previously described [35]. Detailed CTLC sample characteristics are provided in **Supplementary Table S1**.

### 3.2 Gene-by-PAE interaction analyses for neurobehavior and growth outcomes examining child and maternal genetic data separately

In CTLC’s single marker SNP*PAE interaction analyses of child data, one SNP approaching GWAS significance (*rs2833924*, *p*=8.8x10^−8^) was associated with working memory performances, with several additional SNPs with marginal significance (*p* <10^−5^) reported in **Supplementary Table S2**.

In single marker SNP*PAE interaction analyses of maternal data, we found one SNP approaching GWAS significance (*rs17050152*, non-synonymous exonic variant in *SYNPO2*, *p*=7.7x10^−7^) associated with working memory (additional suggestive (*p* <10^−5^) results in **Supplementary Table S2**).

Top findings from gene-based gene-by-PAE analyses of child data identified a significant locus, *STX6* (*p*= 2.2x10^−6^) associated with reduced child height (Figure 2A) and a second locus significant at trend, *FOXD3* (*p*= 4.7x10^−6^), associated with recognition memory performance (Figure 2B). No significant loci were identified in mothers. Suggestive genes (*p* <10^−4^) for working memory, recognition memory, and FAS diagnosis in child and, for working memory, recognition memory and height, in mothers are reported in **Supplementary Table S3**.

### 3.3 Genetic main effects on FAS/PFAS diagnosis (exposed only) examining child and maternal genetic data separately

No significant loci were observed in single-marker nor gene-based analyses in either maternal or child strata for FAS/PFAS diagnosis. However, four genes showed marginal associations (**Supplementary Table S3**). Similarly, in the CIFASD dataset, no SNP or gene reached genome-wide significance, with six single markers and six genes showing marginal significance (**Supplementary Table S4 and S5**).

### 3.4 Meta-analysis of Single-Marker and Gene-Based Association Results (child genetic data only)

Meta-analysis of the CTLC and CIFASD cohorts single-marker association using the Han and Eskin random-effects (RE2) model identified several loci showing suggestive evidence of association across multiple neurocognitive and FASD-related phenotypes. For working memory, the most significant association was observed at locus rs12090855 (chr1:28874969 HG19) (P_RE2=1.38x10^−6^), with additional suggestive signals at loci rs2125398 (chr4:75208519) and rs1990171 (chr7:86885119) (P_RE2 <1x10^−5^. For recognition memory, the strongest association signal was detected at locus rs1048274 (chr9:6256292) (P_RE2=1.65x10^−6^, with additional consistent signals at loci including rs4349637 (chr4:182065498) and rs12805702 (chr11:25117751) (P_RE2 <1x10^−5^ across multiple recognition memory model definitions. Here, P_RE2 is the p-value derived from the Han and Eskin random-effects (RE2) meta-analysis model, which is designed to maintain statistical power under heterogeneous effect sizes across cohorts while accounting for between-study heterogeneity.

For FASD-related diagnostic and postnatal phenotypes, the top association signals were observed at loci rs10105040 (chr8:94711093), rs7866848 (chr9:97505608), and rs946488 (chr1:215415487), with P_RE2 values ranging from 7.65x10^−6^ to 1.20x10^−5^. Notably, these loci were consistently identified in both neurobehavioral diagnostic and postnatal height analyses, suggesting shared genetic influences across FASD-related phenotypes. Finally, no genome-wide results were observed in single-marker analyses. The meta-analysis of gene-based association results found a significant genome-wide association in the *HTR1E* gene associated with working memory performance (*p*=2.75x10^−6^) (Figure 2C).

### 3.5 Haplin Transmission Disequilibrium Test

In CTLC, gene-by-PAE HTDT test found no significant maternal or child haplotypes after multiple testing correction for neurobehavior and growth outcomes. For FAS/PFAS diagnosis, no significant main effect was found.

We followed up multiple nominally significant haplotypes from the genotype array data (**Supplementary Table S6, S7**) employing imputed data: we found three haplotypes showing significant main effects after frequency weighted FDR correction on FAS/PFAS diagnosis (Table 2). A haplotype in children lying within the *COP1* gene (*p_adj_*= 0.0295), and two maternal haplotypes lying within the *WWOX* gene (*p_adj_* = 0.0002) and within an intergenic region between the *TMEM38B* and *MIR8081* genes (*p_adj_* = 0.0192). The risk ratios associated with the significant haplotypes in offspring and mother are shown in Figure 2. Comparison of effect sizes of the significant imputed haplotypes in child and mother showed the opposite direction in the confidence intervals. This regional enrichment of nominally associated haplotypes suggests that these loci harbor underlying association signals that were more effectively captured after imputation due to increased marker density and improved haplotype resolution.

Figure 3 presents the forest plot of relative risk (RR) and 95% confidence intervals for the haplotypes that remained significant after multiple testing correction in the imputed dataset. The Chr1 (C-A-G-A) haplotype showed a protective effect in the offspring genome (RR = 0.19, 95% CI: 0.08–0.44, adjusted p = 0.029), whereas the Chr9 (C-c-g-c) and Chr16 (C-A-C-G) haplotypes showed significant risk effects in the maternal genome, with relative risks of 3.47 (95% CI: 1.87–6.43, adjusted p = 0.019) and 4.07 (95% CI: 2.11–8.11, adjusted p = 2×10^−4^), respectively. These results indicate distinct maternal and offspring haplotype effects at different loci, suggesting that both maternal genetic background and offspring genotype may contribute to FASD-related phenotypes.

### 3.6 Differential gene expression results

Table 3 displays the results of differential gene expression analyses based on PAE status (exposed vs. unexposed) in CTLC for risk-modifying genes described above, with adjustment for tissue-specific cell-type composition and technical confounders (using SVA). The cell type distribution for placenta and child blood is shown in **Supplementary Figure S3**. *HTR1E*, implicated in FAS/PFAS diagnosis, and *FOXD3*, implicated in recognition memory, were not expressed in placenta or child blood. *STX6*, which had evidence of gene*PAE interaction effects on child height, showed increased expression in child blood. For FAS/PFAS diagnoses, *COP1,* which had evidence of child genetic main HTDT effects, showed no differential expression. Two genes with evidence of maternal genetic HTDT main effects exhibited notable patterns: *TMEM38B* showed reduced expression in child blood, and *WWOX* demonstrated decreased expression in both placenta and child blood. Collectively, these transcriptomic findings provide functional support for several genetic signals and highlight tissue-specific regulatory patterns that may contribute to fetal vulnerability to prenatal alcohol exposure.

## 4. DISCUSSION

Integrating child and maternal genetic data from two prospective longitudinal birth cohorts and child genetic data from a clinical cohort of exposed and unexposed children, we identified several novel genetic loci that may modify the risk of FASD. To our knowledge, this is the first FASD study to utilize genome-wide approaches and the first to integrate maternal and child genetics.

We employed single-marker and gene-based approaches and the Haplin Transmission Disequilibrium Test. Our gene-based and Haplin analyses were substantially higher yield than single-marker analyses in identifying potential risk-modifying child and maternal loci, likely due to the reduced multiple testing burden in gene-based analyses; genome-wide screens and gene-by-environment interaction analyses are both very power intensive. It should be noted that a large number of loci were found to have marginal significance in both single marker and gene-based analyses, greatly exceeding standard power estimations. This yield likely reflects the high PAE levels and unique genetic architecture in our cohorts and the potent teratogenicity of alcohol.

The key strength of the Haplin Transmission Disequilibrium Test over standard approaches is that it examines maternal and child genetics simultaneously, thereby disentangling the shared genetics between mother and child. The multiple-testing burden of a haplotype approach (2-3 million haplotypes), and use of binary “case” variables rather than continuous quantitative traits make this approach especially power intensive. Furthermore, as in gene-based approaches, strong gene-by-environment interaction signals may be diluted and thus obscured by markers with weaker effect sizes in the same haplotype/region. Notably, our HTDT examination of main effects of genotype on risk for child FAS/PFAS diagnosis among exposed children revealed one child locus and two maternal loci. The precision of FAS/PFAS diagnostic criteria and the specificity of those diagnoses to PAE likely enhanced power in these analyses.

The detection of significant haplotypes in the imputed dataset but not in the genotype-only dataset is likely attributable to the increased marker density provided by imputation, which improves haplotype reconstruction and enables the capture of untyped variants in linkage disequilibrium with causal loci. Consistent with this, we observed multiple nominally significant haplotypes in the surrounding genomic regions in the genotype dataset, suggesting the presence of underlying regional association signals that became statistically significant only after imputation increased genomic coverage and refined haplotype structure. The significant haplotype signals detected for FAS/PFAS in the imputed CTLC HTDT analysis likely arose because the informative SNPs defining these haplotypes were not present in the directly genotyped CTLC dataset and therefore became analyzable only after imputation. These SNPs and haplotypes were also absent in the CIFASD dataset, limiting replication across cohorts. Additional differences between findings from directly genotyped and imputed datasets may reflect differences in variant coverage, linkage disequilibrium structure, and ancestry composition across cohorts, particularly within African and admixed populations where haplotype diversity is substantial. Such factors can influence the transferability and consistency of association signals across datasets.

In contrast, analyses based on directly genotyped data produced more consistent and interpretable findings across most phenotypes, suggesting that imputation quality and population mismatch were important limiting factors in the imputed dataset. These findings highlight the need for improved population-specific reference panels and optimized imputation strategies when analyzing genetically diverse cohorts such as CTLC.

The child and maternal genetic loci identified in our analyses have remarkable biologic plausibility for contributing to FASD pathophysiology. Child markers included rs2833924, which is in the ‘Down syndrome critical region’ in a binding site for several transcription factors near cytokine genes (*IFNA, IFNB, IL10RB)* relevant for neuroinflammation, which is implicated in FASD; *RUNX1*, which helps regulate hematopoiesis and neurodevelopment [48]; and several genes involved in mitochondrial function, which has also been implicated in FASD [49]. *STX6* mutations are associated with CEDNIK Syndrome, in which children exhibit developmental delay, absence of the corpus callosum, hypomyelination, and white matter abnormalities [50], all of which we have previously demonstrated in FASD [23, 51, 52]. Notably, in our data, *STX6* blood expression was increased among exposed vs. control children. *FOXD3* is down-regulated in response to PAE in animal models and plays critical roles in differentiation of neural crest cells, a key target in alcohol teratogenesis [53, 54]. *HTR1E* encodes the 5-hydryoxygtryptamine-1E G-protein-coupled serotonin receptor, which is highly expressed in the brain and promotes neuronal survival against oxidative and neurotoxic stress [55]. Maternal genetic loci included *COP1*, which encodes a ubiquitin ligase that suppresses neuroinflammation, especially in microglia [56]. Notably, in our data, *COP1* blood expression was increased among children with FAS or PFAS vs. non-syndromal children. Maternal *rs17050152* approached significance and is in *SYNPO2*, a tumor suppressor gene that positively regulates actin filament bundle assembly and cell migration. Alterations in *SYNPO2* expression have been associated with ethanol exposure in adult rodent models and with alcohol use in humans [57, 58]. *TMEM38B* encodes a calcium channel important for Type I collagen synthesis and is maternal blood biomarker for disrupted placental development/preeclampsia [59]. *WWOX* is tumor suppressor gene that is important for placental glucose control/metabolism in mother and neurodevelopment in child [60]; mutations lead to microcephaly, epilepsy, developmental delay, early death [61]. In our data, placental *WWOX* expression was increased in exposed vs. control children. It should be noted that genes not found to be differentially expressed based on PAE or phenotype may still play important roles in the pathophysiology of FASD; they may be differentially expressed in other tissues or in specific developmental time windows not captured in our study design.

A limitation of these analyses is the small sample size for genome-wide approaches. To address this limitation, we employed gene-based approaches to reduce the multiple testing burden. Power boosting strengths of our South African cohorts include the very heavy PAE levels and the unique genetic architecture of participants, who identify as South African Cape Coloured. In data from these cohorts, 29,847 monomorphic variants in the gnomAD European reference population [62] were polymorphic in our cohorts, and 90% of these were *common* (≥1% frequency) in our cohorts. We have also demonstrated a high proportion (>2/3) of non-synonymous (i.e., functionally relevant) exonic variants, unusually high homozygosity (6%), and high proportions of homozygous genetic segments that ancestrally harbor a higher burden of deleterious variants [63]. Future studies are needed to validate/replicate our findings in larger cohorts, with the caveat that most existing large cohorts have lower levels of PAE than our South African cohorts. Differences in findings between our South African cohort and the CIFASD database may be due, in part, to cohort differences such as allele frequencies that vary by geographic ancestry. However, gene-based analyses are less dependent on allele frequencies of specific variants within a gene that may vary across populations. Another possible source of these differences in findings is the different neurobehavioral measures utilized across datasets. Future studies are needed that incorporate multiple domain-specific outcome assessments into latent variables that are comparable across cohorts. Lastly, the MEGA^ex^ array does not provide complete genome coverage, and as noted above, imputation in the South African Cape Coloured population is prone to errors. Thus, our analyses may have missed important genetic variants that were not assayed, such as rare variants that often have large effect sizes. Future studies are needed that utilize whole genome sequencing to capture such variants.

## 5. CONCLUSIONS

In summary, in this genome-wide screen for risk modifying maternal and child genetic loci, we identified several putative genes that may have physiologic significance in the teratogenic effects of alcohol and may inform future work to identify specific markers that identify pregnancies and/or children at risk for FASD. Our findings also highlight the benefits of utilizing multi-tissue transcriptomics in genetics research. Furthermore, our findings support the need for genome-wide approaches in FASD research and underscore the importance of examining both maternal and child genetics when studying FASD and other developmental diseases.

## Supplementary Material

Supplementary information is available at MP’s website.

This is a list of supplementary files associated with this preprint. Click to download.

- GeneticInfluencesFASDMolPsychSuppMatJune052026.pdf
- GeneticInfluencesFASDMolPsychSuppMatJune102026.pdf

## Figures and Tables

**Figure 1.**
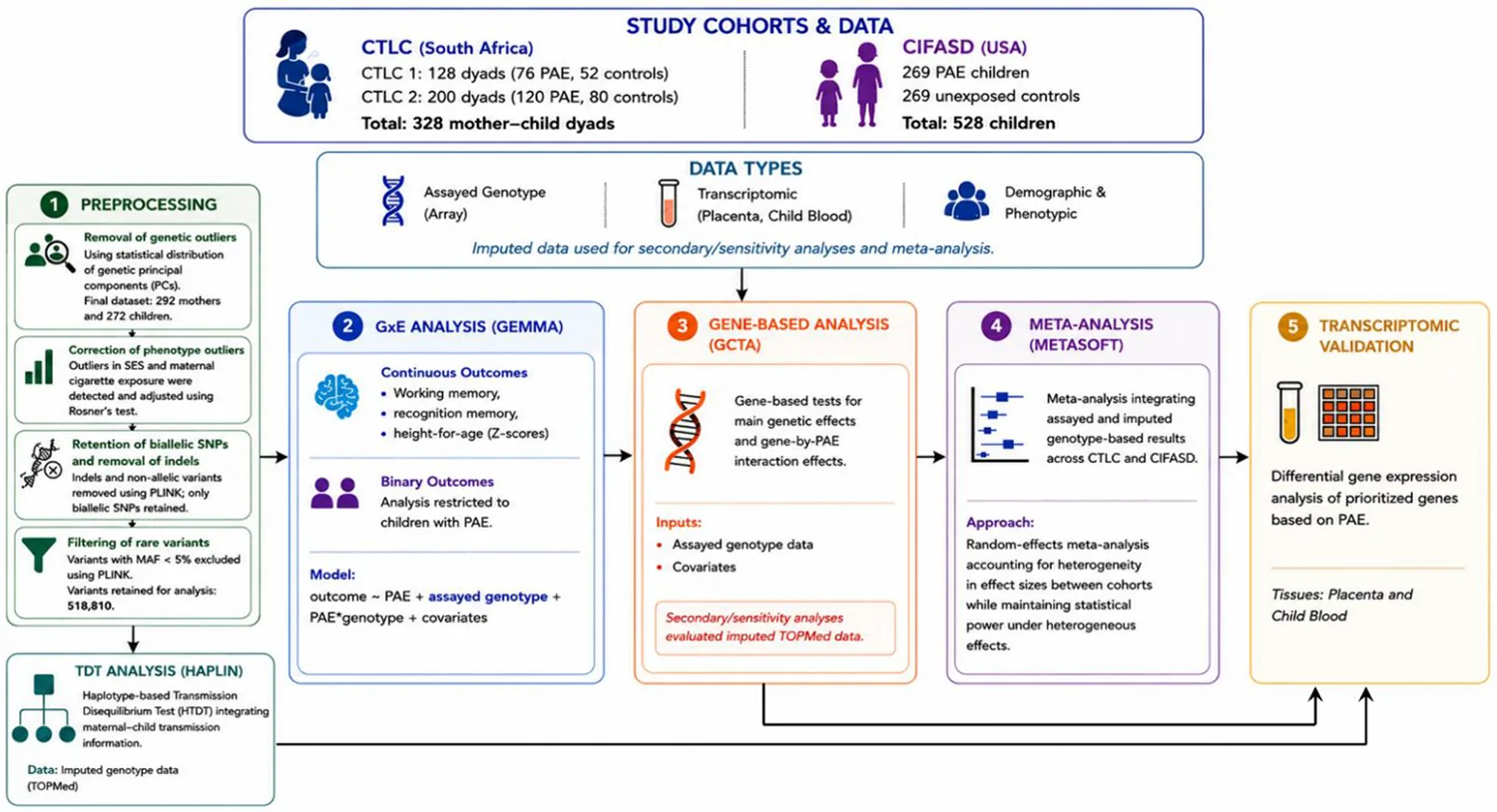
Overview of the study design and analytical workflow for genome-wide investigation of maternal and child genetic factors associated with FASD outcomes. Genotype array, TOPMed-imputed genotype, transcriptomic, demographic, and phenotypic data from the Cape Town Longitudinal Cohorts (CTLC) and Collaborative Initiative on Fetal Alcohol Spectrum Disorders (CIFASD) cohorts were analyzed. Following genotype quality control and preprocessing, gene-by-environment (GxE) interaction analyses were performed using GEMMA, gene-based analyses using GCTA, and cohort-level meta-analyses using METASOFT. Haplotype-based Transmission Disequilibrium Test (HTDT) analyses were conducted in CTLC mother–child dyads using Haplin. Prioritized loci were subsequently evaluated using transcriptomic validation in placenta and child blood datasets.

**Figure 2:**
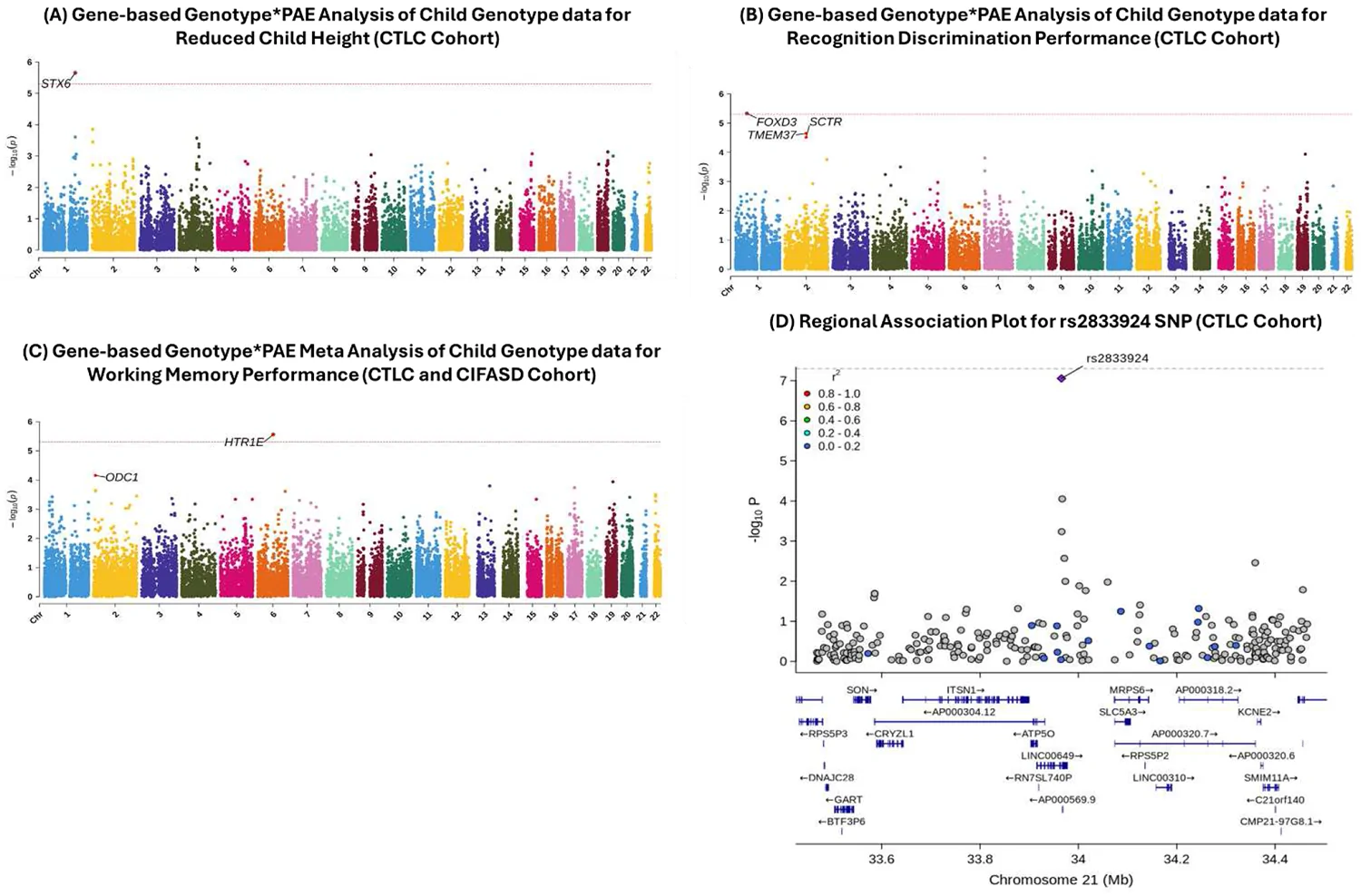
Genome-wide association results (genotype data) for neurobehavioral and growth-related traits in CLTC data. (A) Gene-based association analysis for child height-for-age z-scores adjusted for postnatal covariates and Heavy_YN exposure (λ = 1.075). The Manhattan plot highlights one genome-wide significant gene (*STX6*). (B) Gene-based association analysis for child recognition memory adjusted for neurobehavioral covariates and Heavy_YN exposure (λ = 1.05). Three genes (*FOXD3, TMEM37, CTR*) show marginal associations. (C) Gene-based meta-analysis results for child working memory in CTLC and CIFASD. (D) Regional Association plot for approaching genome-wide significant rs2833924 SNP in working memory performance. The Manhattan plot highlights HTR1E gene as genome-wide significant. The QQ plots for all these Manhattan plots are shown in **Supplementary Figure S2**.

**Figure 3:**
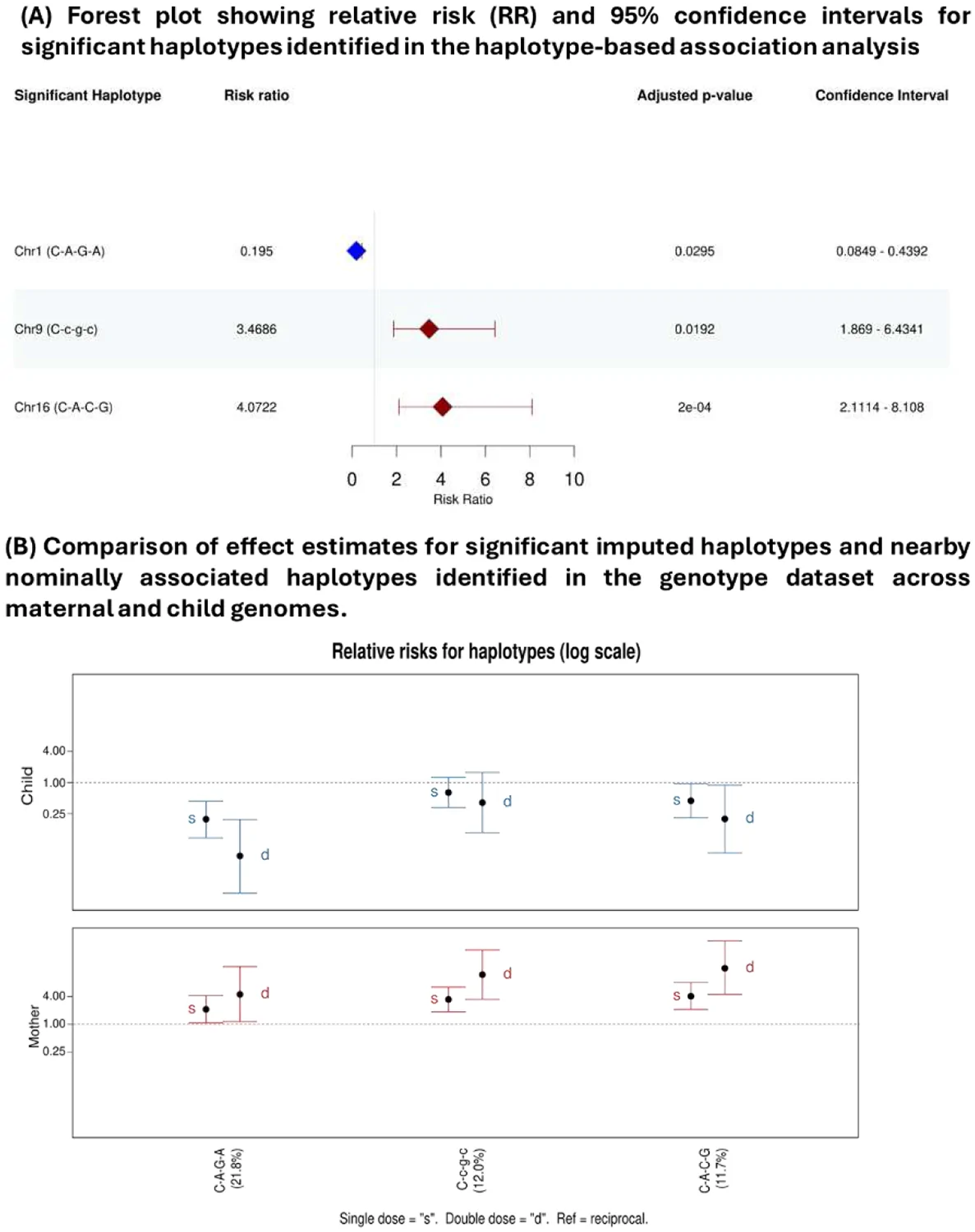
Haplotype association results and relative risk (RR) with 95% confidence interval (CI) for FAS/PFAS diagnosis at Chr1, Chr9, and Chr16 loci. The top panel shows a forest plot of RR and 95% CI for haplotypes that remained significant after multiple testing correction in the imputed dataset. The bottom panel compares the RR of these significant haplotypes in mother and offspring for single (s) and double dose (d). Blue indicates offspring (child) haplotypes and red indicates maternal haplotypes. Error bars represent 95% confidence intervals.

**Table 1.** Sample characteristics in the Cape Town Longitudinal Cohorts

		Controls *M* (SD) / n (%)	Heavy drinking mothers *M* (SD) / n (%)	*p* ^a^
N		132	196	
Maternal characteristics				
Maternal age at conception (yr)		25.84 (5.23)	27.33 (6.07)	0.018
Education (yr school completed)		9.88 (1.75)	8.71 (2.22)	**< 0.001**
Socioeconomic status score ^b^		22.82 (7.64)	19.84 (7.67)	**0.006**
Alcohol consumption	oz AA/day	0.0 (0.0)	0.90 (1.1)	**< 0.001**
	oz AA/drinking day	0.10 (0.35)	4.20 (2.42)	**< 0.001**
*n* (%) reporting cigarette smoking		80 (60.6)	169 (86.22)	**< 0.001**
Cigarettes/day among smokers		6.04 (5.40)	7.11 (4.71)	0.1293
Child characteristics				
Weeks gestation at delivery		38.97 (2.05)	38.69 (2.05)	0.2437
Infant sex, no. female (%)		65 (49.24)	100 (51.02)	0.7531
FAS or PFAS diagnosis, *n* (%)		0 (0)	72 (36.73)	**< 0.001**

FAS = fetal alcohol syndrome using Hoyme 2005 criteria [6]; PFAS = partial FAS.

a From independent samples t-tests for continuous variables and chi-squre for categorical variables.

**Table 2.** Genetic haplotypes with main effects on FAS/PFAS diagnoses (imputed data)

Haplotype	OUTCOME	Risk Ratio (95% CI)	Haplotype Frequency	adjusted *p*	Function	Gene
CHILD						
chr1:175992527:T:C chr1:175992552:G:A chr1:175992588:G:C chr1:175992852:A:G	FAS/PFAS	0.19 (0.08–0.44)	0.22	0.0295	Intronic	*COP1*
MOTHERS						
chr9:109190024:C:T chr9:109190198:G:C chr9:109190646:C:G chr9:109191014:G:C	FAS/PFAS	3.47 (1.87–6.43)	0.12	0.0192	Intergenic	*TMEM38B; MIR8081*
chr16:78493391:T:C chr16:78493396:T:A chr16:78493458:C:G chr16:78493612:A:G		4.07 (2.11–8.11)	0.1170	0.0002	Intronic	*WWOX*

a Haplin Transmission Disequilibrium Test

**Table 3.** Differential gene expression^a^ based on alcohol exposure (yes vs. no) for potential risk-modifying genes

			Placenta		Child blood	
Outcome	Gene prioritized by GWAS	Strata	logFC	*p*	logFC	*p*
Height	*STX6*	Child	0.3124	0.2131	0.7480	***0.0230***
FAS/PFAS	*COP1*	Child	−0.00549	0.9761	0.2641	*0.4729*
	*TMEM38B*	Mother	−0.9751	0.211	−1.397	***0.0129***
	*WWOX*	Mother	−0.7567	**0.0358**	−0.8289	***0.0500***

N.E. = not expressed.

a Differential gene expression expressed as log(fold-change) [FC]
